# Supplementary material for: High-Sensitivity Troponin I and Creatinine Kinase-Myocardial Band in Screening for Myocardial Injury in Patients with Carbon Monoxide Poisoning
Source: Diagnostics (Basel). 2020 Apr 21;10(4):242. doi: 10.3390/diagnostics10040242 (PMC7235999; doi:10.3390/diagnostics10040242)
Supplement: Supplementary file 1 [file diagnostics-10-00242-s001.pdf]

Supplementary Table S1. Ability of cardiac biomarkers to predict CO-induced LV dysfunction.

**Cardiac Markers**	**Total (*n*= 850)**	**No LV Dysfunction (*n* = 761)**	**LV Dysfunction (*n* = 89)**	***p***
Initial CK-MB (ng/mL)	1.50 (0.60–5.70)	1.60 (0.60–5.20)	24.2 (9.4–60.3)	< 0.01
Peak CK-MB (ng/mL)	1.80 (0.60–8.65)	1.90 (0.70–6.00)	31.35 (13.80–73.60)	< 0.01
Initial hsTnI (ng/mL)	0.01 (0.01–0.16)	0.02 (0.01–0.13)	1.41 (0.38–3.16)	< 0.01
Peak hsTnI (ng/mL)	0.03 (0.01–0.39)	0.03 (0.01–0.27)	1.82 (0.67–4.90)	< 0.01
BNP (pg/mL)	18.00 (6.0–56.00)	15.00 (6.00–43.00)	78.50 (28.00–242.00)	< 0.01

Abbreviations: CO = carbon monoxide; LV = left ventricle; CK-MB = creatinine kinase-myocardial band; hsTnI = high-sensitivity troponin I; BNP = brain natriuretic peptide.

Supplementary Table S2. Ability of cardiac biomarkers to predict CO-induced RV dysfunction.

**Cardiac Markers**	**Total (*n* = 850)**	**No RV Dysfunction (*n* = 829)**	**RV Dysfunction (*n* = 21)**	***p***
Initial CK-MB (ng/mL)	1.50 (0.60–5.70)	1.80 (0.60–7.90)	31.80 (13.05–60.25)	< 0.01
Peak CK-MB (ng/mL)	1.80 (0.60–8.65)	2.30 (0.70–11.10)	35.40 (20.90–89.20)	< 0.01
Initial hsTnI (ng/mL)	0.01 (0.01–0.16)	0.03 (0.01–0.24)	2.36 (0.56–4.64)	< 0.01
Peak hsTnI (ng/mL)	0.03 (0.01–0.39)	0.04 (0.01–0.42)	2.36 (0.68–8.88)	< 0.01
BNP (pg/mL)	18.00 (6.00–56.00)	17.00 (6.00–53.00)	112.00 (48.50–306.00)	< 0.01

Abbreviations: CO = carbon monoxide; RV = right ventricle; CK-MB = creatinine kinase-myocardial band; hsTnI = high-sensitivity troponin I; BNP = brain natriuretic peptide.

Supplementary Table S3. Ability of cardiac biomarkers to predict CO-induced WMA.

**Cardiac Markers**	**Total (*n* = 850)**	**No WMA (*n* = 788)**	**WMA (*n* = 62)**	***p***
Initial CK-MB (ng/mL)	1.50 (0.60–5.70)	1.70 (0.60–5.70)	29.70 (10.20–76.70)	< 0.01
Peak CK-MB (ng/mL)	1.80 (0.60–8.65)	2.10 (0.70–8.05)	33.90 (20.700–97.90)	< 0.01
Initial hsTnI (ng/mL)	0.01 (0.01–0.16)	0.02 (0.01–0.16)	1.63 (0.45–3.26)	< 0.01
Peak hsTnI (ng/mL)	0.03 (0.01–0.39)	0.03 (0.01–0.32)	1.95 (0.69–5.94)	< 0.01
BNP (pg/mL)	18.00 (6.00–56.00)	16.00 (6.00–46.50)	114.50 (53.00–294.00)	< 0.01

Abbreviations: CO = carbon monoxide; WMA = wall motion abnormality; CK-MB = creatinine kinase-myocardial band; hsTnI = high-sensitivity troponin I; BNP = brain natriuretic peptide.
